# Medical Reminiscences of New York*Explanatory Note.—The writer wishes to state that he cannot vouch for the accuracy of these memoranda, in all respects. They were written during his recent sojourn in New-York City in this manner: He wrote off at night *what he recollected* of what he saw and heard *of interest* during the day. If any inaccuracies occur, therefore, whether of expression, statement or opinion, let them be ascribed to the writer, and not to the gentlemen whose teachings are sketched.

**Published:** 1860

**Authors:** D. C. O’Keefe

**Affiliations:** Atlanta, Ga.


### ARTICLE V.

*Medical Reminiscences of New York.** By D. C. O'KEEFE, M. D., Atlanta, Ga.

### PROF. BEDFORD'S CLINIQUE.

*Case* 1st.—A child the subject of ascarides. There is no *sure* sign of the presence of worms but seeing them after being voided. This child's mother says she has seen them in the patient's evacuations, and therefore it may be regarded a case of "worms." The thread worm inhabits the rectum, and sometimes gives rise to much irritation, tenesmus and itching at the anus, and sometimes a mucous discharge from the vagina or urethra.

*Treatment.*—Administer a local remedy to kill the worms, and then give a purgative to carry them off. ℞—Lime water, two ounces; muriated tincture of iron, half an ounce. Inject half this into the rectum at night, the remaining half next morning; then give a dose of castor oil to carry them off.

*EXPLANATORY NOTE.—The writer wishes to state that he cannot vouch for the accuracy of these memoranda, in all respects. They were written during his recent sojourn in New-York City in this manner: He wrote off at night *what he recollected* of what he saw and heard *of interest* during the day. If any inaccuracies occur, therefore, whether of expression, statement or opinion, let them be ascribed to the writer, and not to the gentlemen whose teachings are sketched.

*Case* 2.—Stomatitis in a child one to two years old. There are four varieties of the disease: simple erythematic, do. with curd-like exudation, follicular and ulcerative. Treatment—chlorate of potash internally—may be given with the child's nourishment, and with decided benefit.

*Case* 3.—Paraplegia in a child two years old—may be produced by cold, sudden fright, such as confinement in a dark closet or room for purposes of punishment; intestinal irritation, &c., &c. In this case the paraplegia is consequent on an attack of acute enteritis—a not uncommon sequence of that disease, as first pointed out by that inimitable observer, Dr. Graves, of Dublin. Treatment—Improve the general health. Prescribed quinine, tincture of hyosciamus, dilute sulph. acid and water.

*Case* 4.—Obstinate constipation in a woman 20 years old —went as long as a month sometimes without an evacuation. Treatment—Mercurials, followed by sulphate magnesia.

*Case* 5.—Carcinoma uteri in a woman about 40 years old —was presented two weeks ago, at which time she was suffering from hemorrhage from vagina which had existed five months, but which has ceased from the application of potassa cum calce made at that time. Treatment—Give the liquor arsenicalis to relieve pain—sometimes a very good remedy, and then again it has no effect. At next clinic will apply the potassa cum calce again, which is better than the potassa fusa.

*Case* 6.—Hemorrhage from vagina produced by submucous fibrous tumor of the uterus. These vaginal discharges are not original diseases, only symptomatic—the smoke from the fire. They arise from carcinoma, fibrous tumors, cauliflower excrescence, inflammatory inturgescence, polypus, &c., &c. This case has existed for three years. The treatment of this woman is twofold—palliative and radical; will adopt the former for the present, and give the tincture of secale cornutum. An operation would accomplish the radical treatment.

PROF. METCALFE'S CLINIQUE.

*Case* 7.—Chronic bronchitis. There are no rales present in this case; respiration weak; expectorates freely. Do not give expectorants when the expectoration is free and easy already. Give this man tonics.

*Case* 8.—Hemeplegia in an old man presented at last clinique—told us then he (the Prof.) had no faith in medicines in such cases; when cures occur, nature affects them; says he feels better than he did one week ago, but the improvement should not be attributed to the remedies used; was probably getting better when he first presented himself; he suffered from constipation, for which aloes and strychnia were prescribed—thought his constipation depended on paralysis of the muscular coat of the small intestines, over which strychnia has a good influence.

*Case* 9.—Emphysema of 14 years standing—of which the man will never recover; has also varicose veins of the legs, the result of the same. (*Query.*—What is the rationale of it?") He has also latent pleurisy with effusion on one side, as indicated by dullness, bronchophony, &c., &c. Treatment—Diureties and iodide of potassium to get rid of the effusion.

MISCELLANEOUS CLINIQUE BY PROF. CLARK.

*Case* 10.—Patient, a man aged about 40; a case of difficult diagnosis—has had seven different opinions pronounced on it, the last of which ("I don't know") is the most sensible. He has worked with paints eighteen months, and has symptoms of lead poisoning, Burtons line, &c. Has stridulous cough and breathing, prodnced, *perhaps*, by the pressure of an aortic aneurism on the recurrent laryngeal nerve which supplies the larynx. The external jugulars are very full and prominent. Diagnosis not made out.

*Case* 11.—Neuralgia of left shoulder and arm in a female. There is an enlargement on the upper surface of the first rib, but nothing wrong about the chest. Osteal enlargement from mercury was thought to be a common disease in the last generation. He (Dr. C.) had not seen much of it—did not know whether it was a correct opinion or not. Treatment—Citrate of iron and quinine—is disposed to regard this treatment a test as to whether it is neuralgia or not.

*Case* 12.—Onanism in a male youth. His heart beats tumultuously—nothing else abnormal about it but intensified action. Treatment—Hands off; quit the habit.

BELLEVUE HOSPITAL—SERVICE OF PROF. METCALF.

*Case* 13.—Autopsy at Dead-House. Subject, a man aged 45 years; was sick but six weeks, (by his own account) and died of carcinoma of the liver, which weighed 12 pounds.—It presents a nodulated appearance, with cup-shaped cavity in each nodule—this cup in the knot is pathognomonic of cancered liver. This form of hepatic disease can be distinguished during life, by its knotty feel from engorgement, fatty degeneration, hydatids, scirrhus, &c., of that organ.—It has also undergone fatty degeneration discoverable only by the microscope.

*Case* 14.—Bright's disease of the kidney, of three year's duration, in a young man aged 20, by occupation a bar-keeper. He cannot see a wink—has amaurosis, a part and parcel of the disease. Has dropsy of the face, abdomen and extremities—that of the face is pathognomonic of the disease. His mouth bleeds, and he is altogether a miserable looking object (a powerful argument against intemperance). Treatment—Sustain him; there is no cure for him.

*Case* 15.—Aneurism of aorta. Symptoms present dullness over upper sternal region; impulse of tumor felt just above sternum by deep pressure with the ends of the fingers into the fossa in that region; bulging of upper sternal region; absence of respiratory murmur in left lung from pressure of tumor on bronchus—puerile respiration in other lung; great hoarseness—lost his voice at one time, but has partly recovered it again; prominence and fullness of jugular veins.

The hoarseness and aphonia are the result of pressure on recurrent laryngeal nerve. Treatment—Nothing can be done.

*Case* 16.—Chronic diarrhœa. Patient extremely emaciated; has no chest symptoms—has, in all probability, abdominal phthisis. Had cured a case of chronic diarrhœa last year, with Carb. Magnesia, paregoric and water—died afterwards of phthisis. Treatment—Subnitrate of bismuth. One week afterwards an autopsy of this case was had at the Dead-House, with the following results: Two large ulcers in colon near cœcum—these ulcers are probably tuberculous, which can only be ascertained by microscopic examination; enlargement of solitary glands—the ulcers of cancer and tubercle are generally transverse, (across the gut); those of typhoid fever are longitudinal (along track of gut); thickening and softening of parts of mucous coat; a tubercular condition of the mesenteric glands—are full of cheesy matter; there are indurated tubercles in the lungs, and the liver is in a fatty condition (yellow). Chronic diarrhœa, or chronic dysentery, is nearly always owing, in this city, to one of three causes, viz: Cancer, tubercle, or typhoid fever. It is a frequent disease in warm climates, and many of the troops who served in the Mexican war, suffered from it.

*Case* 17.—Ozœna in a young man 24 years old. There was contraction (narrowing) of one nostril. Had symptoms of masturbation also. Said he had not practiced it since he was 16 years old—doubtful; said he had *never* indulged in sexual intercourse. This portion of his case, therefore, he (Dr. M.) regarded as one of chronic virtue. Treatment for Ozœna—Throw a pint syringe full of a detergent solution up the nostril of the affected side, first directing the patient to take a deep inspiration, and hold his breath so as to close the opening of the glottis, and prevent the fluid from getting into the larynx. Repeat the injection two or three times a day, which in combination with an alterative will cure the case. (Two other cases of masturbation were seen in the wards. I believe the morbific effects of this habit are over-estimated—that many of them have their foundation in the imagination, induced by the perusal of the writings of quacks).

*Case* 18.—Probable softening of the brain from some kind of tumor: subject, an Englishman aged about 40, a hatter by trade. Has been in the hospital but a few days—there is a loss of consciousness and of motion; has had severe pain in one side of his head for two years, and within two weeks has had several attacks of epilepsy.

PROF. CLARK'S CLINIQUE.

Several specimens of urine were presented, having a thick crust formed on top, after standing from one week to several months. This crust is called the penicilium glaneum, and composed of vegetable matter eliminated from the body by the kidneys. These particles are the result of dis-assimilation of the food, unassimilated particles of which get into the circulation, and circulate in the blood, acting as a poison. The original fault of this imperfect assimilation is in the ganglionic system. It gives rise to various symptoms; such as giddiness, pains in various parts of the body, depression of spirit, &c., &c.

*Case* 19.—Probable neuralgia of side of head and face of ten days standing, in connection with three wounds of scalp. It may be connected with the wounds, or it may be neuralgia. If the latter, 20 grains cit. iron and quinine a day will cure it in a few days.

*Case* 20.—A young man who has been doctored by a quack for tape-worm, and told by him that he had voided some. On being shown a specimen of the genuine worm from the museum, he declared that what he had passed had no resemblance to it. His symptoms are epileptiform; thinks he has no tape-worm; its passage is the only evidence of its existence. You cannot know when the whole of the worm is expelled until you see its head, which is towards the stomach, and is the last part voided. Its neck is thin and attenuated; its head has four suckers and a disk which require a magnifying glass to see them; is composed of joints $\frac{1}{8}$ to $\frac{1}{2}$ inch long; is very rare in persons born in the United States—

more common in Germans and Dutch, from eating . . . . . pork. Treatment of epilepsy—Sulphate or oxide of zinc.

*Case* 21.—Ascites in a woman 40 years old. Her abdomen is flabby and tympanitic; legs œdematous. Diagnosis—cirrhosis of the liver, which is small, as shown by percussion. The œdema does not belong to this condition of liver, except as a result of general cachexia, from which this woman does not suffer, for her face is full and ruddy; it is owing probably to Bright's disease of the kidney, which cannot be ascertained without testing the urine for albumen. The spleen is also a good deal enlarged, probably the result of former malarial influences—not essential in the case. If this enlarged abdomen were from ovarian or uterine tumors, it would not be so flabby—there would be hardness felt. Treatment—If the kidneys be not diseased, use diuretics in connection with diaphoretics and purgatives; if they be, omit the diuretics and use the other two; the warm bath does great good in cases like this. Related the case of a lady at Williamsburg in which the warm bath had a most happy effect.

*Case* 22.—Cachexia in a child 2 to 3 years old from cardiac disease. Has been taking iron and is better, but will never be well.

*Case* 23.—The case of suspected aneurism (case 10) of aorta, presented at last clinique, appeared to-day, and the diagnosis was pronounced aneurism. It afforded a fine illustration of cavernous respiration, the rationale of which is this: the tumor presses on the trachea and obstructs the passage of air, which, when it passed the seat of pressure into the large open space below it, gave rise to a sound like that of air entering a cavity.

Bellevue Hospital—service of Prof. Metcalf—Postmortem at Dead-House. Subject, died of delirium tremens. No organ particularly diseased. The mode of death seemed to be from asthenia—a failure of the heart's action; the nervous system is poisoned from continued drink and succumbs. The brain was nearly healthy—slight congestion of arachnoid.

*Case* 24.—Pneumonia in case of delirium tremens seen at last visit. This is extremely common here—we do little for it; if you treat it much, they die, and they are apt to die any how.

*Case* 25.—Pneumonia in German young man seen at last visit; was very sick then; had double pneumonia; is much better now; pulse 80-90; doing very well now. This case has had but little treatment; doubt whether it exercised any curative agency whatever; the disease has a strong tendency to self-cure, if not interfered with; therefore, don't be surprised at the homeopaths curing it. Almost every case with a tolerably good constitution gets well in this hospital on little or no treatment. Bleeding does no good; may relieve dyspnœa and pain by lessening the quantity of blood sent to the lungs, but only for a few hours. Has bled but three patients with pneumonia in all his practice, public and private. Calomel does no good; only makes the mouth sore.*

*Case* 26.—Delirium tremens—in straight jacket, after a spree of four weeks; has had it a week; is in a kind of stupor. He (Dr. M.) believes these cases would get well without treatment; put them where they can do no harm, feed them well and let them rip; if they don't sleep for several nights, it don't matter; they will sleep when they get tired of staying awake.

*Case* 27.—Abscess of liver or hydatids of same in a young man—impossible to tell which; quite a large abdominal tumor in hypogastric region; seemed to point somewhat. An ulcer had been made with the vienna paste on the most

*NOTE.—These doctrines on pneumonia will, doubtless, startle some, as they did the writer, but he believes they will be found correct by those who adopt them in practice. Dr. Fleishman, of Vienna, lost but six per cent. on the globule treatment; whilst Dr. Dictl, of the same city, treated 189 cases on the strictly expectant plan—put them to bed and dieted them, but gave them *no* medicine, and lost seven per cent. The difference of one per cent. in favor of homeopathy may find a ready explanation in errors in diagnosis and the desire natural to a new system of making a favorable exhibit. How many of us cure 93 or 94 out of every 100 of our cases of pneumonia?

prominent point of the tumor, with the view of inducing adhesions between the peritoneal covering of the liver and the walls of the abdomen, and thus prevent the escape of matter into the peritoneal sack. This may also be a case of encephaloid disease of the liver, but from the history of the case, did not think so. It might seem that fluctuation would distinguish between abscess and encephaloid disease; but he had seen the most experienced men so often mistaken that he believed it was a very unreliable test; had heard Dr. Mott say he had given up trying to distinguish between the fluctuation of an abscess and the feel of an encephaloid tumor.

*Case* 28.—Aneurism of aorta in a man aged 25-30, very fat and thickly made; has cough, owing, doubtless, to bronchitis, dyspnea and a peculiar blowing or stridulous breathing, from which, alone, he would suspect the existence of anuerism. This stridulous breathing is produced by pressure on the trachea or bronchus; there is very strong impulse felt under the hand applied over top of sternum; indeed, the walls of the chest are seen in motion from the impulse; no impulse felt in jugular fossa by burying the fingers therein; no pulse in radial artery of right side; signs of congestion in cervical veins; respiration peurile under left clavicel; wanting in right lung, front and back; dullness over space of 4 inches long and 2½ wide at top of sternum. The aneurism is probably in the ascending aorta before it arches, and presses on right bronchus; has had rheumatism, syphilis and gonorrhœa. Sometimes these aneurismal tumors destroy bones and other tissues that impede their progress.*

*NOTE.—The writer has seen, at Demilt Dispensary, in the service of that most accomplished auscultator, Dr. Chamman, a striking example of the destruction of tissues effected by anuerismal tumors. On the 25th August I have this note: Dr. Camman called our attention to the case of anuerism of the aorta which he had presented at the Dispensary once or twice. The subject of it was an Irishman about 40 years old, a shoemaker by trade. He had an aneurism which projected externally at the top of the sternum as large as a child's head, involving the absorption of the top of the sternum, the sternal extremeties of both clavicles and of one or two ribs on each side. The integument over its apex was very thin from absorption of the tissues covering it, and was in momentary danger

*Case* 29.—Gonorrhœal ophthalmia in a man 35 years old. The expression of the face in this disease is characteristic: the lids are very much swollen, eyes firmly closed and can hardly be opened; and when the lid is lifted, a free discharge of yellow or dark looking pus gushes out. It is nearly always associated with gonorrhœa, and you should never fail to ascertain that fact, so that the nature of the ophthalmia may be certainly known. It often destroys vision, and will do so in this case, probably, in one eye. Treatment—Paint the inflamed eye-balls with the stick of nitrate of silver twice a day until the inflamed surface looks white; use astringent collyria to wash out the purulent discharge very often; (every half hour, Dr. Gouley says;) leech freely, &c., &c.*

---

of bursting and killing the patient instantly. To retard this event as long as possible, it was kept covered with a piece of sheet lead accurately fitted to it, which, by its pressure, might induce inflammation in the integument, and thickening of the same by a deposit of fibrin. He directed attention to its great size, and the man's immunity from any inconvenience from it --no dyspnœa; whilst 'in another case he had shown us an aneurism of the same parts, where there was no external tumor, and where its presence was ascertained by the physical signs; the patient suffered a great deal from dyspnœa. This was accounted for by the direction the tumors took. In the first case the tumor was developed outwards; there was no pressure on the trachea, and therefore no dyspnœa; but in the second case, the tumor, though a small one, grew directly backwards, pressed on the trachea, and hence the dyspnœa. This man died suddenly a few days ago, as was expected. On rising in the morning, he found the tumor bleeding, called for assistance and died before any one reached him. On postmortem examination, the aneurism was found as diagnosed, and involving also the arteria inominata. Pieces of the sternum and ribs were found in the tumor.

*NOTE.—There is at this time a case of the same disease in the female surgical ward, service of Dr. Gouley, in a young woman in whom no trace of gonorrhœa can be found. She is a washerwoman, and the supposition is, that she contracted the disease from infected clothing.)

*Case* 30.—Rapia (large scabs)—a form of tertiary syphilis —more pleasant to treat than to have.

